# Global Patterns and Climatic Controls of Dust-Associated Microbial Communities

**DOI:** 10.1128/Spectrum.01447-21

**Published:** 2021-10-13

**Authors:** Yongjian Chen, Matthew J. Gebert, Seth A. Faith, Robert R. Dunn, Noah Fierer, Albert Barberán

**Affiliations:** a Department of Environmental Science, University of Arizona, Tucson, Arizona, USA; b Department of Ecology and Evolutionary Biology, University of Colorado, Boulder, Colorado, USA; c Cooperative Institute for Research in Environmental Sciences, University of Colorado, Boulder, Colorado, USA; d Department of Microbiology, The Ohio State University, Columbus, Ohio, USA; e Department of Applied Ecology, North Carolina State University, Raleigh, North Carolina, USA; University of Minnesota

**Keywords:** aerobiology, allergens, climate change, dust microbiomes

## Abstract

The ubiquity and long-range transport of the microorganisms inhabiting dust can pose a serious risk to human, animal, and plant health. The well-recognized importance of dust-associated microorganisms contrasts starkly with our limited understanding of the factors determining the variation in the composition of these communities at the global scale. Here, we provide the first insight into the global determinants of dust-associated microorganisms by quantifying the environmental factors shaping bacterial and fungal community composition in 467 outdoor settled dust samples collected from 33 countries and 6 continents. Our results show that the global variation in dust-associated bacterial and fungal community composition was, to some degree, predictable from mean annual precipitation and temperature. Notably, our results show that the fungal genera *Alternaria* and Aspergillus, which contain many species that can serve as triggers of allergenic disease in humans and as plant pathogens, were more abundant in drier regions. Collectively, these results highlight the key influence of climate on the global distribution of dust-associated microorganisms and provide the baseline information needed to build a more comprehensive understanding of how microbial exposures vary across the globe and in response to climate change.

**IMPORTANCE** A broad diversity of microorganisms can be found in dust, with some of these microorganisms capable of causing allergenic disease in human via inhalation or affecting plant health by acting as plant pathogens. However, the spatial variation in dust microbiomes and the environmental factors associated with this variation have not been comprehensively assessed at the global scale. Here, we investigated the bacteria and fungi found in outdoor settled dust samples spanning 33 countries and 6 continents. Our results show that dust-associated bacteria and fungi exhibit climate-driven variability in community composition at the global scale. Our results call for the development of strategies to predict the geographic distribution of dust-associated microorganisms and to identify the potential associations between microbial exposures and the health of humans, animals, and plants.

## INTRODUCTION

In dust and outdoor air, microorganisms are ubiquitous and diverse, and they can represent a large fraction of aerosolized particles (1). Exposure to these microorganisms can have detrimental effects on human, animal, and plant health (2). For example, allergic asthma, which is often triggered by exposure to airborne microorganisms (3), affects more than 300 million people worldwide, and it is estimated to account for approximately 1,000 deaths per day (4). Moreover, dust-dwelling plant pathogens represent a major threat to agricultural production and food security (5). Furthermore, transoceanic and transcontinental dust events can transport microorganisms thousands of kilometers away from their sources, resulting in pronounced ecological disturbances to distant sink ecosystems (6). Although dust-associated communities of microorganisms have been extensively studied using both cultivation-dependent and cultivation-independent approaches, with these methods yielding important insights into the types of bacteria, fungi, and other taxa that can be found in dust (7–10), few studies have directly investigated how the composition of microorganisms varies across large spatial scales and the environmental factors associated with this variation.

Geographic variability in the composition of dust-associated microorganisms can arise from differential contributions of source environments. Dust settled on external surfaces is considered to be a long-term reservoir of microorganisms that were previously airborne and can reenter the atmosphere through dust resuspension (11). Emissions from a variety of source environments, such as soils, waterbodies, leaf surfaces, humans, and animals, can serve as important contributors to the microbial loading of dust and outdoor air (12). Previous studies have shown that the relative importance of different source environments can vary across seasons, land-use types, and vegetation types (13–16). For example, soil-associated microorganisms are more abundant in the air above forests and agricultural landscapes than in suburban areas (14). In addition, geographic variability in dust-associated microbial community composition can track the changes in environmental attributes. A survey of settled dust collected outside homes across the United States demonstrated that the composition of bacterial and fungal communities varied as a function of climatic conditions and soil properties (17). Likewise, climatic seasonal changes modulate the temporal dynamics of airborne microbial communities, with cold-tolerant and ice-associated microorganisms predominant during snowy seasons (13). However, the majority of previous studies have only focused on studying the environmental drivers of community variability in dust microbiomes at the local or regional scales. Our systematic and comprehensive understanding of dust-associated microorganisms at the global scale remains limited. As we know that the abundances of some microorganisms associated with human diseases (e.g., allergic disorders) can vary across geographic regions (18), a global investigation of the environmental factors shaping the distribution of outdoor dust-associated microorganisms is crucial for understanding the potential impacts of climate change and other global change factors on public health.

**FIG 1:**
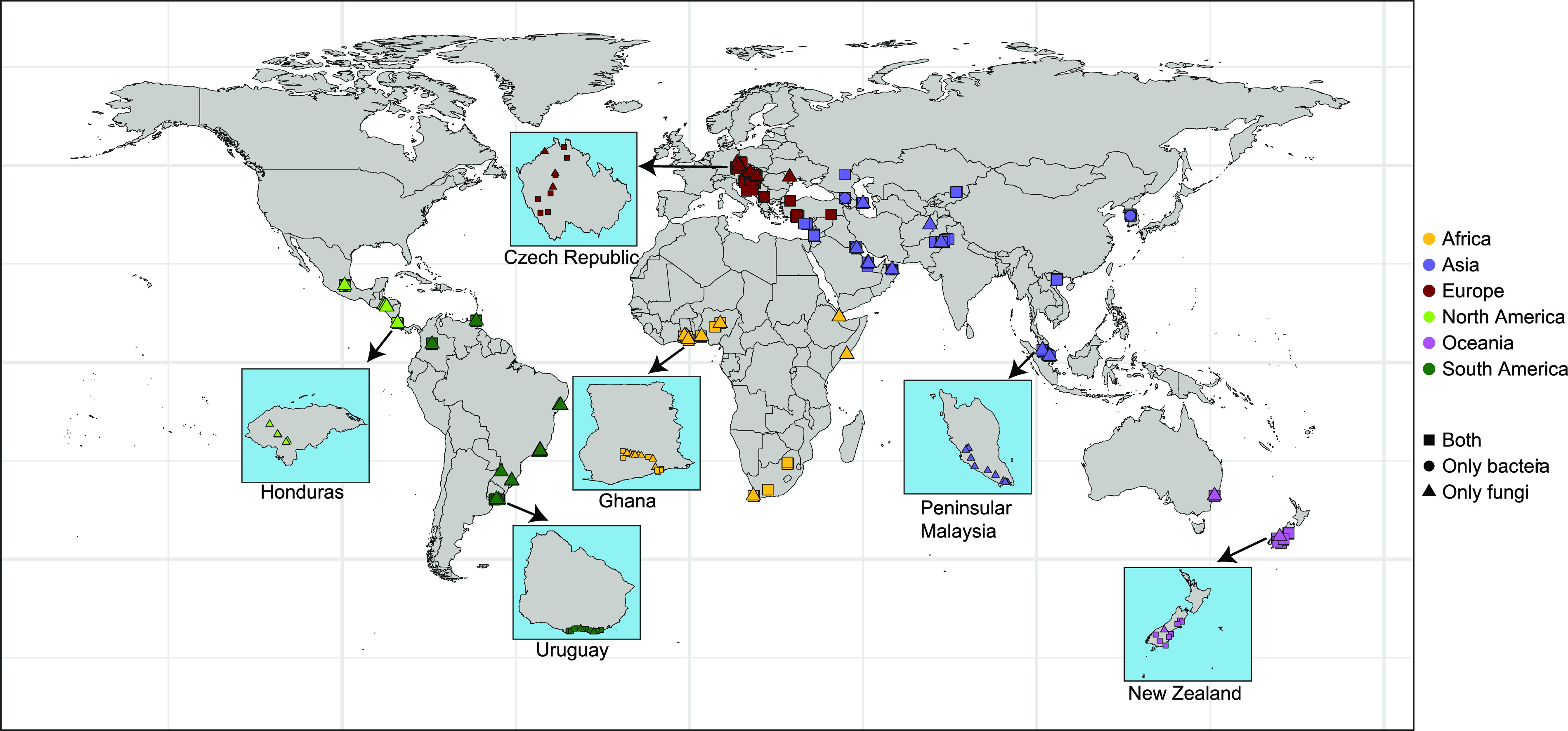
Geographic distribution of dust samples. A total of 467 samples of dust that had accumulated on outdoor surfaces were collected from 33 countries and 6 continents. The squares denote samples having both bacterial and fungal data, the circles denote samples having only bacterial data, and the triangles denote samples having only fungal data. For each continent, a higher resolution map of the sampling locations in one sample country is shown. See Fig. S1 for all the types of external surfaces.

Here, we conducted a global survey of outdoor dust settled on external surfaces. The dust samples were collected by swabbing various external surfaces such as window sills, door trim, walls, and fences. A total of 467 dust samples were collected from 33 countries and 6 continents (Fig. 1 and Fig. S1) (19), representing the largest attempt to comprehensively investigate the global distribution of outdoor dust-associated microbial communities. The bacterial and fungal communities in dust samples were characterized by sequencing the V4 region of the 16S rRNA gene and the first internal transcribed spacer region (ITS1) of the rRNA operon, respectively. Because our study was conducted at the global scale, we were primarily interested in understanding how broad-scale environmental factors determine the global variation in dust-associated microbial communities. Specifically, our aims were to (i) identify the key climatic or soil variables predicting the global variation in the community composition of outdoor dust-associated microorganisms and (ii) reveal the associations between climatic or soil variables and the relative abundances of globally prevalent microbial lineages, especially those that are potentially linked to public health.

## RESULTS

### General description of global dust microbial communities.

We obtained a total of 1,813,789 bacterial and 3,861,464 fungal sequences. These sequences were assembled into a total of 13,492 (415 ± 260, mean ± standard deviation [SD]) bacterial and 18,870 (88 ± 51, mean ± SD) fungal phylotypes (Fig. S2 and S3). We found that 42.23% of bacterial phylotypes and 87.10% of fungal phylotypes were restricted to one continent (Fig. S4). Likewise, 96.16% of bacterial phylotypes and all fungal phylotypes were detected in fewer than half of the samples (Fig. S5A and S5B). At the genus level, 91.83% of bacterial genera and 99.10% of fungal genera were found in fewer than half of the samples (Fig. S5C and S5D).

At the class level, the bacterial communities were dominated by *Alphaproteobacteria* (23.29%), *Actinobacteria* (16.64%), *Bacilli* (8.37%), *Cytophagia* (7.48%), and *Gammaproteobacteria* (6.13%). The fungal communities were dominated by *Dothideomycetes* (42.91%), *Eurotiomycetes* (12.42%), *Tremellomycetes* (7.74%), *Cystobasidiomycetes* (6.81%), and *Agaricomycetes* (6.79%). At the genus level, there were on average 106 bacterial genera (range: 13 to 303) and 47 fungal genera (range: 3 to 153) detected per sample. The top 10 most abundant bacterial genera were *Hymenobacter*, *Paracoccus*, *Microbispora*, *Rubellimicrobium*, *Sphingomonas*, *Janthinobacterium*, Acinetobacter, *Chroococcidiopsis*, *Rubrobacter*, and *Kaistobacter* (Fig. S6A). The dominant fungal genera were Aspergillus, *Alternaria*, *Cladosporium*, *Symmetrospora*, *Naganishia*, *Nigrospora*, *Aureobasidium*, *Pseudopithomyces*, *Knufia*, and *Toxicocladosporium* (Fig. S6B). The relative abundances of these genera were highly variable across the global dust samples (Fig. S6). For example, the relative abundances of the fungal genera Aspergillus and *Alternaria* in individual samples ranged from 0 to 85.27% and 0 to 68.62%, respectively (Fig. S6B).

### Environmental determinants of microbial communities.

**FIG 2:**
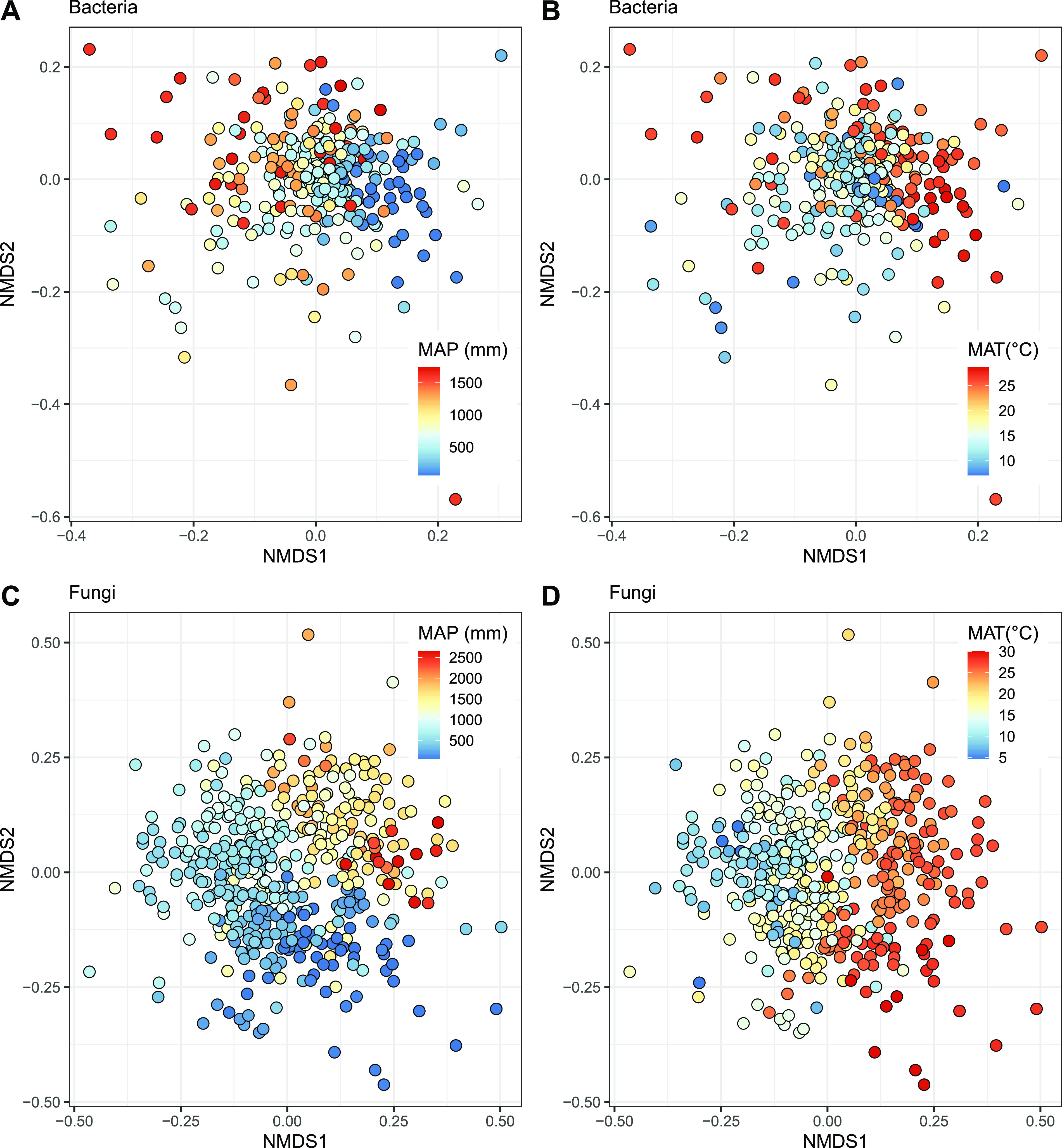
Environmental determinants of microbial community composition. NMDS ordination plots show the changes in bacterial (stress, 0.21) (A and B) and fungal (stress, 0.23) (C and D) community composition along gradients in mean annual precipitation and mean annual temperature (for both, *P = *0.001 in MRM models). MAP, mean annual precipitation; MAT, mean annual temperature.

Stochastic factors contributed 82.52% and 79.41% of the variation in bacterial and fungal community composition, respectively. Despite the large impact of stochastic factors, the global variation in bacterial and fungal community composition was, to some degree, predictable from environmental factors. The results of variation partitioning analyses showed that environmental factors explained a larger fraction of variation in microbial community composition than geographic distance (Table S1). For bacterial communities, multiple regression on distance matrices (MRM) showed that global variation in community composition was best predicted by mean annual precipitation (5.57%, *P = *0.001), and to a lesser extent, mean annual temperature (3.57%, *P = *0.001), geographic distance (2.64%, *P = *0.001), and precipitation of the driest month (2.43%, *P = *0.001). For fungal communities, MRM showed that the strongest driver of community composition was mean annual temperature (7.03%, *P = *0.001), followed by mean annual precipitation (5.08%, *P = *0.001) and geographic distance (3.96%, *P = *0.001). These patterns are evident from the nonmetric multidimensional scaling (NMDS) ordination plots in Fig. 2, which illustrate how dust-associated bacterial and fungal community compositions shift across gradients in mean annual precipitation and temperature.

**FIG 3:**
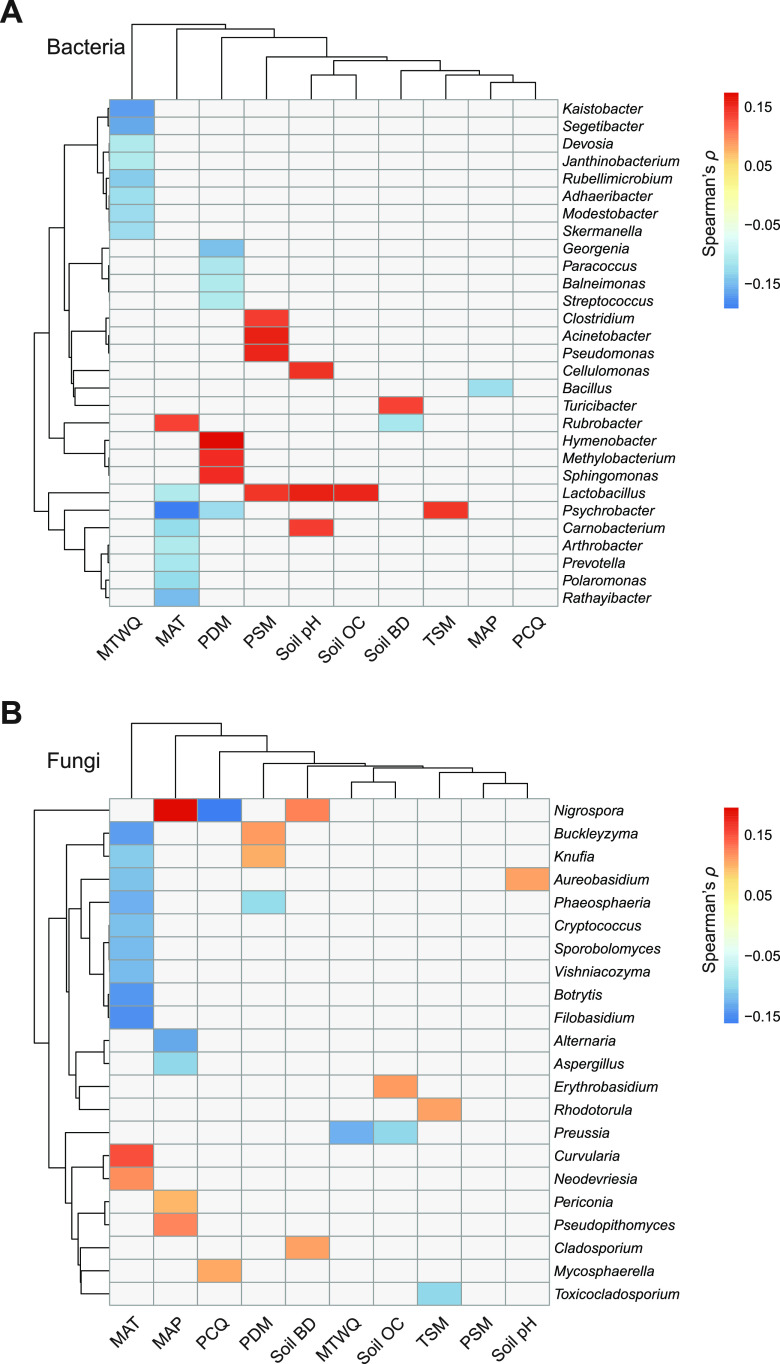
Environmental determinants of the relative abundances of the prevalent genera of bacteria (A) and fungi (B). Only those genera significantly correlated with at least one environmental variable are shown (semipartial Spearman rank correlation, *P < *0.05). The dendrograms show the results of hierarchical cluster analysis, which was conducted based on the statistically significant semipartial Spearman rank correlation coefficients. MAT, mean annual temperature; MTWQ, mean temperature of the wettest quarter; TSM, temperature of the sampling month; MAP, mean annual precipitation; PDM, precipitation of the driest month; PCQ, precipitation of the coldest quarter; PSM, precipitation of the sampling month; soil OC, soil organic carbon; soil BD, soil bulk density.

**FIG 4:**
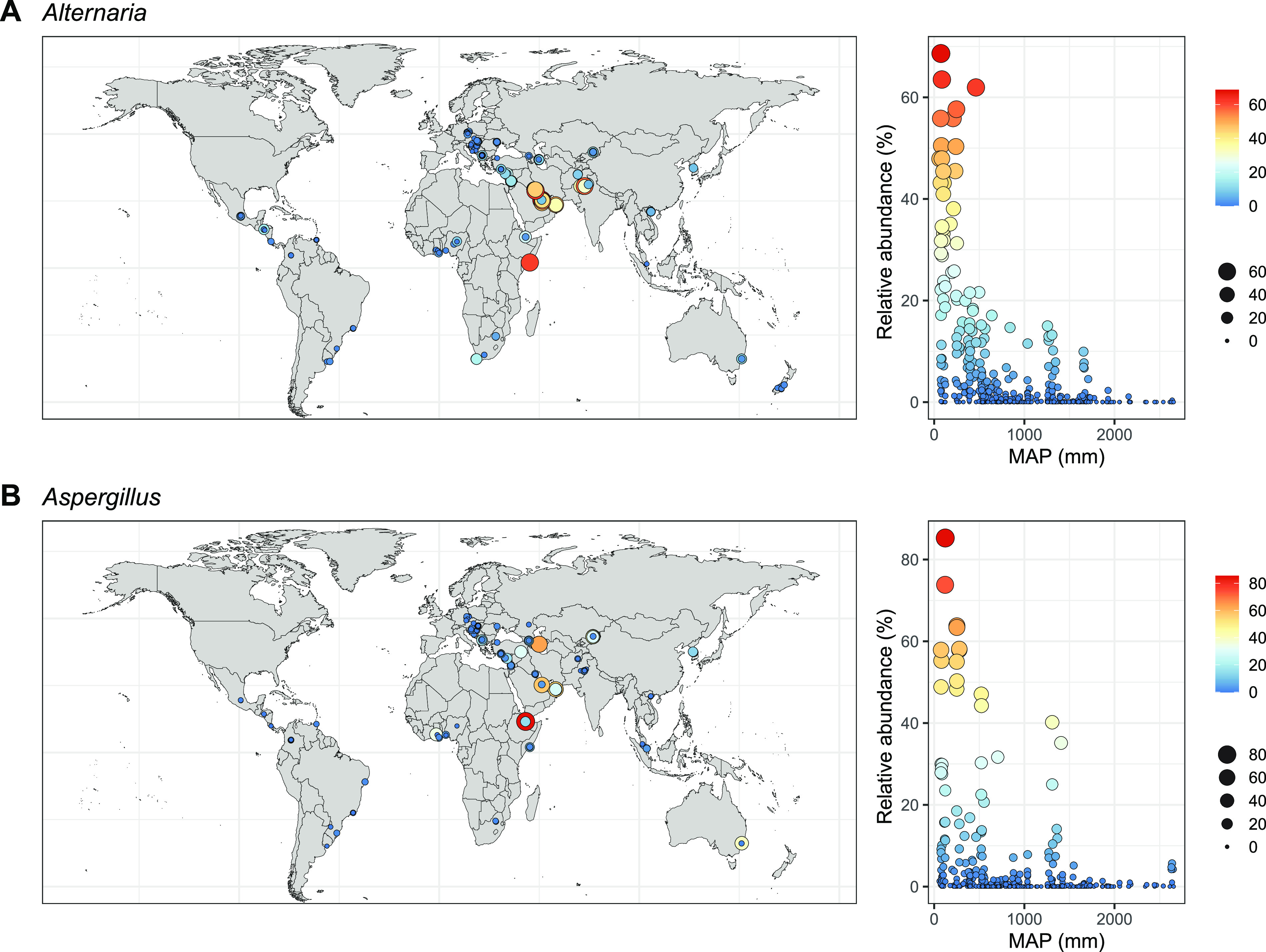
Geographic patterns of the relative abundances of *Alternaria* (A) and Aspergillus (B). The color of the points indicates the relative abundance, and the point sizes are proportional to the relative abundance. Samples with zero relative abundance of either *Alternaria* or Aspergillus are omitted from the world map, but they are retained in the scatterplot. The relative abundances of both *Alternaria* (Spearman’s *ρ* = −0.42, *P < *0.001) and Aspergillus (Spearman’s *ρ* = −0.26, *P < *0.001) were negatively associated with mean annual precipitation (MAP).

We identified 64 bacterial genera and 32 fungal genera that were prevalent in our global dust samples (Fig. S5C and S5D). The relative abundances of 7 bacterial genera were negatively associated with mean annual temperature (e.g., the cold-climate taxa, *Psychrobacter* and *Polaromonas*; Fig. 3A). The relative abundances of *Sphingomonas* and *Hymenobacter* were positively associated with precipitation of the driest month, and the relative abundance of *Cellulomonas* was positively related to soil pH (Fig. 3A). Notably, the relative abundances of two prevalent and dominant fungal genera, *Alternaria* and Aspergillus, were negatively associated with mean annual precipitation (Fig. 3B and Fig. 4). Both *Alternaria* and Aspergillus were particularly abundant (representing up to 60 to 80% of ITS sequences) in dust samples collected from drier regions in the Middle East (Fig. 4).

## DISCUSSION

### Associations between climatic factors and the global variation in microbial community composition.

Ecologists have long been intrigued by the widespread distribution and high diversity of microorganisms in outdoor dust (20). However, compared to the vast body of research on the biogeography of microorganisms in habitats such as soil and seawater, microorganisms inhabiting dust represent one of the least-studied microbial components of the biosphere (21). Dust-associated microbial community composition can be homogenized by long-distance atmospheric transport and the complexities of local wind patterns (2). These dispersal mechanisms might lead to nonexistent geographical patterns and pose a daunting challenge to identifying the environmental determinants of microbial community variation. Indeed, some of the taxa that we detected were present on every continent. Our results show that global variation in dust-associated bacterial and fungal community composition was, to some degree, predictable from climatic conditions. These global results are consistent with a continental-scale study that showed how the geographic pattern of microbial community composition in outdoor settled dust was associated with climatic variables (17). However, a large part of the observed variation in microbial community composition was unexplained by the included climatic variables. The unexplained variation could be attributable to unmeasured, but important, factors such as surrounding vegetation composition, land-use type, or wind currents. Moreover, there could be stochastic factors controlling the types of microorganisms found in dust, which is confirmed by our findings that stochastic factors dominantly explained the community variability. For example, the presence of dead insects in one sample might lead to dominance by insect-associated bacteria and fungi (17). Despite the large amount of unexplained variation, we found that mean annual precipitation and temperature were the two most important variables associated with differences in bacterial and fungal community composition across the compiled dust sample set. Overall, our results indicate that dust-associated microorganisms exhibit nonrandom and climate-driven variability in community composition at the global scale.

Climatic conditions may determine the outdoor dust-associated microbial community composition in three interrelated ways. First, climatic conditions may shape the composition of microbial communities in the surrounding source environments. Dust-associated microorganisms can be aerosolized from a myriad of nearby source environments such as soils and waters, with climatic conditions directly or indirectly determining the microbial community composition in these potential source environments. For example, the global soil bacterial and fungal community composition is associated with mean annual precipitation and temperature (22, 23), and the global community patterns of marine bacteria are driven by mean annual temperature (24). Therefore, the selection and filtering effects of climatic conditions on microbial communities in source environments could leave an imprint on dust-associated microbial communities. Second, climatic conditions govern how dust-associated microorganisms are aerosolized and transported from source environments. Emissions from source environments require a reduction in the forces holding the microorganisms to the surfaces of the source environments (i.e., bonding effect [25]). This bonding effect is influenced by the moisture and temperature at the surfaces, which can be related to precipitation and temperature regimes (26). For example, drier and warmer conditions can facilitate the aerosolization and dispersal of soil- and plant-associated microorganisms (27). Therefore, climatic conditions can modulate the relative contributions of different source environments to dust-associated microbial communities. And third, differences in climatic conditions could contribute to the variation in the longer-term preservation of microbial taxa in dust. For example, in drier and warmer areas, spore-forming microorganisms are more likely to be persistent in dust settled on surfaces for a longer time due to their capability to resist desiccation and UV radiation (28).

### Associations between climatic factors and the relative abundances of prevalent genera.

For bacteria, our global study identified some of the prevalent genera commonly found in outdoor airborne and dust surveys. For example, it has been shown that the genus *Sphingomonas* is highly abundant in dust settled on balconies (29), and the genus *Hymenobacter* is dominant in the atmosphere (30). In our study, both *Sphingomonas* and *Hymenobacter* were dominant and prevalent genera, and the relative abundances of both taxa were positively associated with the precipitation of the driest month, indicating that they were more common in regions that were more predictably wet. In addition, the relative abundances of the bacterial genera *Psychrobacter* and *Polaromonas* were negatively associated with mean annual temperature. These results are expected because *Psychrobacter* and *Polaromonas* are well known to contain many psychrophilic microorganisms commonly found in low-temperature environments (31), and previously, *Psychrobacter* was found to dominate airborne bacterial communities in winter (13). Furthermore, the relative abundance of the bacterial genus *Cellulomonas* was positively related to soil pH, which is consistent with a previous study showing that pH is a significant predictor of the continental-scale distribution of *Cellulomonas* (17).

Our results show that two fungal genera, *Alternaria* and Aspergillus, were more abundant in drier regions. Both *Alternaria* and Aspergillus are frequently identified fungi in dust surveys. For example, these genera have been shown to dominate the fungal community in atmospheric particulate matter (32) and in dust settled on patios (8). Consistent with these studies, our global investigation shows that *Alternaria* and Aspergillus were dominant and ubiquitous taxa in outdoor settled dust. Furthermore, our results show that dry regions in the Middle East were hot spots of *Alternaria* and Aspergillus. It has been suggested that the spores of *Alternaria* and Aspergillus require dry weather to become airborne and disseminate (33, 34). Moreover, given that *Alternaria* and Aspergillus species are frequently found in soils in high abundance (35, 36), it is possible that drier conditions facilitate the aerosolization of soil-associated *Alternaria* and Aspergillus to the near-surface atmosphere and dust. Indeed, dust storms carry a large proportion of *Alternaria* and Aspergillus species that originate from arid soils and transport them to downwind terrestrial and aquatic environments (37, 38). The information about the environmental preferences of *Alternaria* and Aspergillus species and the geographic variation in their relative abundances is noteworthy because these two genera are critical to the health of humans, crops, and livestock (39). Specifically, both fungal genera are well known to contain many species acting as triggers of allergenic disease (e.g., asthma and hay fever) that pose a serious risk to human health and well-being (40). In addition, they contain a myriad of virulent plant pathogens that affect the health and productivity of many economically important crops (e.g., maize, corn, cotton, wheat, and citrus) and plants that are essential food sources for livestock and wildlife animals (41). The high relative abundances of *Alternaria* and Aspergillus species in dry regions raise significant concerns about the potential impact of climate change on human health and agricultural productivity, especially as aridity and dryland areas are anticipated to increase under projected climate change scenarios (42).

### Caveats and conclusions.

To our knowledge, this is one of the more comprehensive studies to document the types of bacteria and fungi found in outdoor settled dust and how their global distributions are associated with climatic conditions. However, it is worth noting that there are important gaps in the geographic coverage of our study. Dust samples from Europe and western Asia were overrepresented, while dust samples from eastern Asia, Africa, North America, South America, and Oceania were scarce. We also want to acknowledge that our analyses did not include local-scale factors such as nearby vegetation, construction activity, and vehicle and pedestrian traffic that could play important roles in shaping the dust microbiomes at finer spatial scales. Future work studying dust-associated microorganisms from understudied geographic regions and incorporating factors operating at local, regional, and continental scales is required to better understand the determinants of dust-associated microbial communities.

We found that the composition of dust-associated bacterial and fungal communities changed predictably along global climatic gradients. Our findings that two putatively pathogenic genera (i.e., *Alternaria* and Aspergillus) were more abundant in drier regions call for future studies identifying the potential associations between their geographic distributions and disease outbreaks. Our analytical approaches can only be used to assess the relative abundances of microbial taxa, and thus, an important next step is to estimate the absolute abundances of individual taxa by pairing quantitative molecular approaches with sampling methodologies that control for the amount of dust collected. Furthermore, although our study focused on bacterial and fungal communities, viruses also represent a highly diverse group of dust-associated microorganisms that pose a major threat to human health (43). Recent work has also shown that dust acts as a reservoir of antibiotic resistance genes (28). Therefore, future studies that focus on how the environment shapes the geographic distribution and composition of dust-associated viruses and antibiotic-resistant microorganisms/genes will provide a more comprehensive understanding of the impacts of microbial exposures on public health.

## MATERIALS AND METHODS

### Sample collection.

From November 2016 to February 2017, a total of 467 outdoor settled dust samples were collected from 33 counties and 6 continents (Fig. 1 and Fig. S1) (19). SecurSwab DUO-V DNA collectors (Bode Technology, Lorton, VA, USA) were used to collect outdoor dust settled on various external surfaces such as window sills (150 samples), door trims (46 samples), walls (35 samples), and fences (30 samples) (see Fig. S1 for all types of external surfaces). These external surfaces serve as passive collectors of outdoor dust and aerosols. However, we do not know how long the dust had accumulated on the surfaces, and we cannot exclude the possibility that some microorganisms are growing on the surfaces themselves (44). The SecurSwab DUO-V DNA collectors were rotated slowly and moved back and forth across the surfaces during sampling. The SecurSwab DUO-V DNA collectors were used to prevent sample loss or contamination when the dust samples were transported back to the laboratory. All dust samples were stored desiccated at room temperature and were sent to the University of Colorado for molecular analyses.

### Molecular analyses.

DNA from each dust swab was extracted using the Qiagen PowerSoil DNA extraction kit (Qiagen, Hilden, Germany) according to the manufacturer’s instructions. To characterize the bacterial and archaeal communities, the V4 hypervariable region of the 16S rRNA gene was amplified using the 515-F (GTGCCAGCMGCCGCGGTAA) and 806-R (GGACTACHVGGGTWTCTAAT) primer pair (45). To characterize the fungal communities, the first internal transcribed spacer (ITS1) region of the rRNA operon was amplified using the ITS1-F (CTTGGTCATTTAGAGGAAGTAA) and ITS2 (GCTGCGTTCTTCATCGATGC) primer pair (46). The primers included the appropriate Illumina adapters, with the reverse primers also having an error-correcting 12-bp barcode unique to each sample to permit the multiplexing of samples. PCRs were performed in duplicate in a final mixture volume of 25 μl, including 12.5 μl Promega Hot Start master mix, 10.5 μl PCR-grade water, 1 μl PCR primers (forward and reverse combined at a 10-μM concentration), and 1 μl extracted genomic DNA. The PCR thermocycler conditions for both the 16S rRNA gene and ITS region consisted of 94°C for 3 min, 35 cycles of 94°C for 45 s, 50°C for 1 min, 72°C for 1.5 min, followed by a final elongation step for 10 min at 72°C. Extraction blanks and negative controls without a DNA template were included in each batch of PCRs to check for possible contamination. The PCR products were cleaned and normalized using the SequalPrep 96-well normalization plate kits (Thermo Fisher Scientific, Waltham, MA, USA). The purified PCR products from all samples were pooled in equimolar concentrations and were sequenced on a MiSeq instrument (Illumina, San Diego, CA, USA). The sequencing was conducted at the University of Colorado Next Generation Sequencing Facility.

### Sequence processing.

The raw reads were demultiplexed using idemp (https://github.com/yhwu/idemp). Then, the raw reads were processed using DADA2 (47), which can resolve exact biological sequences by assembling reads into error-corrected amplicon sequence variants (hereafter, “phylotypes”). The DADA2 pipeline included quality filtering, modeling of the error rate, dereplication, phylotype inference, merging of the paired-end reads, construction of a phylotype count table, chimera removal, and taxonomy assignment. There were three major differences between the DADA2 pipelines for the 16S and ITS reads. First, the length of the ITS region is highly variable, and thus, Cutadapt (48) was used to remove primer sequences prior to quality filtering. Second, during quality filtering, the 16S reads, but not the ITS reads, were truncated to the same length. This is because some ITS variants might be shorter than the truncation length. Third, taxonomic identities were determined using the RDP classifier (49), trained on the SILVA nonredundant (nr) version 132 database (50) for 16S rRNA phylotypes and the UNITE database (51) for the ITS rRNA phylotypes. Those 16S phylotypes without a bacterial domain assignment and assigned to chloroplast or mitochondrial origin were removed. Those ITS phylotypes without a fungal domain assignment were removed. To remove potential contaminants, phylotypes present in the extraction or PCR blanks were removed. We retained samples with more than 1,000 sequences. The final analyses included 302 samples with both bacterial and fungal data, 11 samples with only bacterial data, and 154 samples with only fungal data. The sequence counts were normalized using a cumulative-sum scaling (52).

### Compilation of environmental variables.

We compiled a set of climatic and soil variables from public databases using the geographic coordinates of the dust samples. Specifically, we compiled 19 climatic variables and the temperature and precipitation of the sampling months from the WorldClim database (53). We compiled four soil variables from the Harmonized World Soil database (54) to represent the soil physical and chemical properties. A list of these environmental variables is provided in Table S2.

### Identification of prevalent genera.

To identify the genera that were widespread in our global dust samples (we identified prevalent genera instead of phylotypes because there were too few globally prevalent phylotypes; see Fig. S5A and S5B), we selected the bacterial genera that occurred in more than 50% of the samples (55) and the fungal genera that occurred in more than 30% of the samples (35). We focused on these more widespread genera, given their ubiquity and because it would have been difficult to model the distributions of taxa that occurred in small numbers of samples.

### Statistical analyses.

Statistical analyses were implemented in R (56). The contribution of stochastic factors to microbial community variability was estimated following reference 57. A total of 10 multicollinearity-free variables were retained (variance inflation factor, <5). These environmental variables included seven climatic variables (mean annual temperature, mean annual precipitation, mean temperature of the wettest quarter, temperature of the sampling month, precipitation of the driest month, precipitation of the coldest quarter, and precipitation of the sampling month) and three soil variables (pH, organic carbon, and bulk density). The best subset of environmental variables accounting for the variation in community dissimilarity (Bray-Curtis metric) was selected by the bioenv procedure within the vegan package (58). MRM was used to fit the microbial community dissimilarity as a function of environmental variables and geographic distance (59). The relative importance of explanatory variables was estimated using the relaimpo package (60). Variation partitioning was used to decompose the variation in the microbial community composition into fractions explained uniquely and jointly by environmental factors and geographic distance. The associations between the relative abundances of the prevalent genera and environmental variables were examined using semipartial Spearman rank correlation (61), which allowed us to examine the relationships between the relative abundances of specific genera and particular environmental variables while controlling for other environmental variables. Statistically significant correlation coefficients (*P < *0.05) were used to group the prevalent genera using hierarchical cluster analysis (the “complete” method).

### Data availability.

The data supporting the conclusions of this article are available in Figshare (https://doi.org/10.6084/m9.figshare.14213519).
