# Supplementary material for: Global Patterns and Climatic Controls of Dust-Associated Microbial Communities
Source: Microbiol Spectr. 2021 Oct 13;9(2):e01447-21. doi: 10.1128/Spectrum.01447-21 (PMC8515940; doi:10.1128/Spectrum.01447-21)
Supplement: SUPPLEMENTAL FILE 1 — Supplemental material. Download SPECTRUM01447-21_Supp_1_seq4.pdf, PDF file, 3.3 MB [file spectrum01447-21_supp_1_seq4.pdf]

Supplemental Material for

Global patterns and climatic controls of dust-associated microbial communities

Yongjian Chen[1]*, Matthew J. Gebert[2,3], Seth A. Faith[4], Robert R. Dunn[5], Noah Fierer[2,3], Albert Barberán[1]

[1]Department of Environmental Science, University of Arizona, Tucson, AZ 85721, USA

[2]Department of Ecology and Evolutionary Biology, University of Colorado, Boulder, CO 80309, USA

[3]Cooperative Institute for Research in Environmental Sciences, University of Colorado, Boulder, CO 80309, USA

[4]Department of Microbiology, The Ohio State University, Columbus, OH 43210, USA

[5]Department of Applied Ecology, North Carolina State University, Raleigh, NC 27695 USA

***Corresponding author:**

Yongjian Chen (chenyj@email.arizona.edu)

This PDF file includes:

Figures S1 to S6

Table S1 and S2

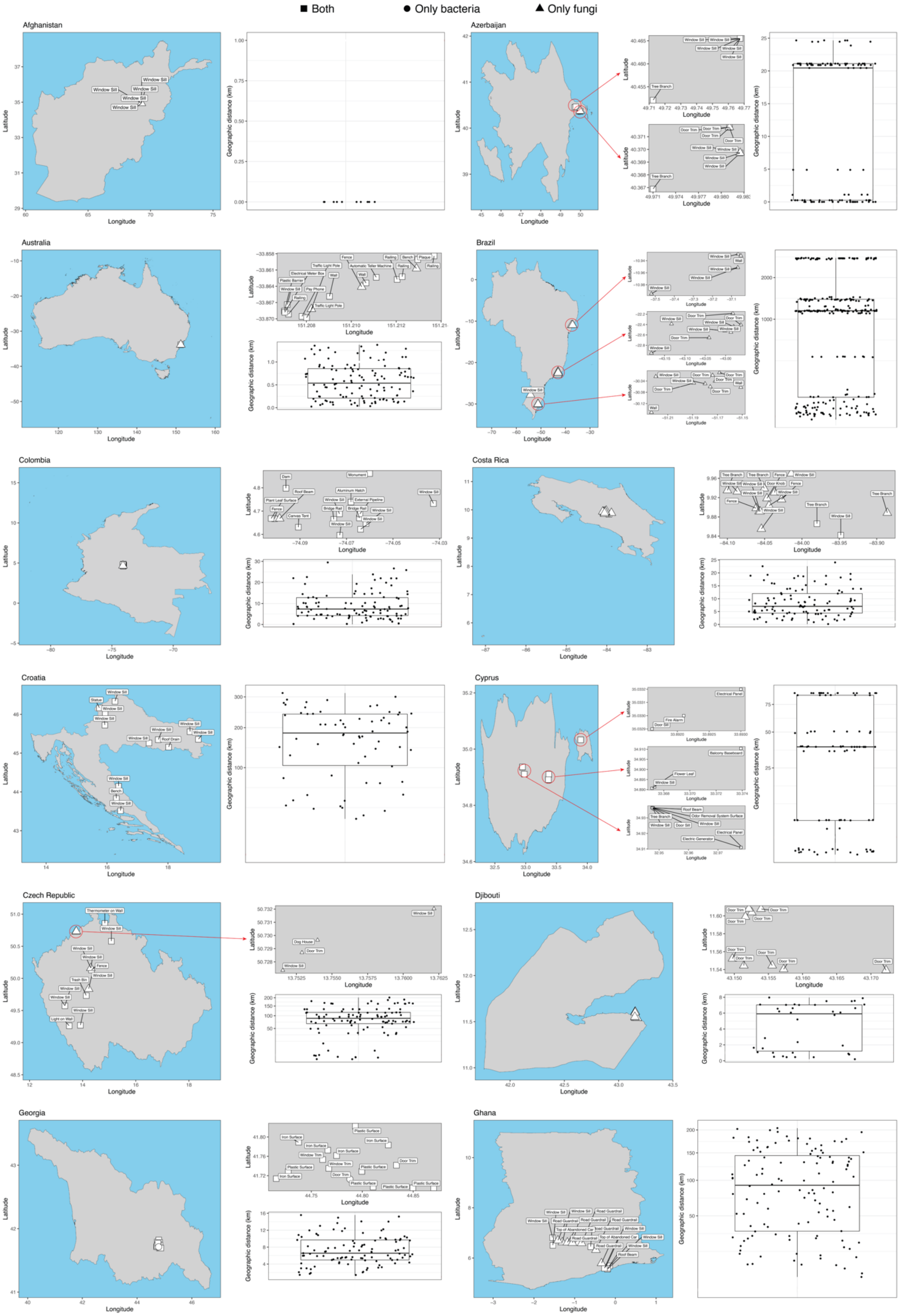

Both
Only bacteria
Only fungi
Afghanistan
Azerbaijan
Australia
Brazil
Colombia
Costa Rica
Croatia
Cyprus
Czech Republic
Djibouti
Georgia
Ghana
Latitude
Longitude
Geographic distance (km)

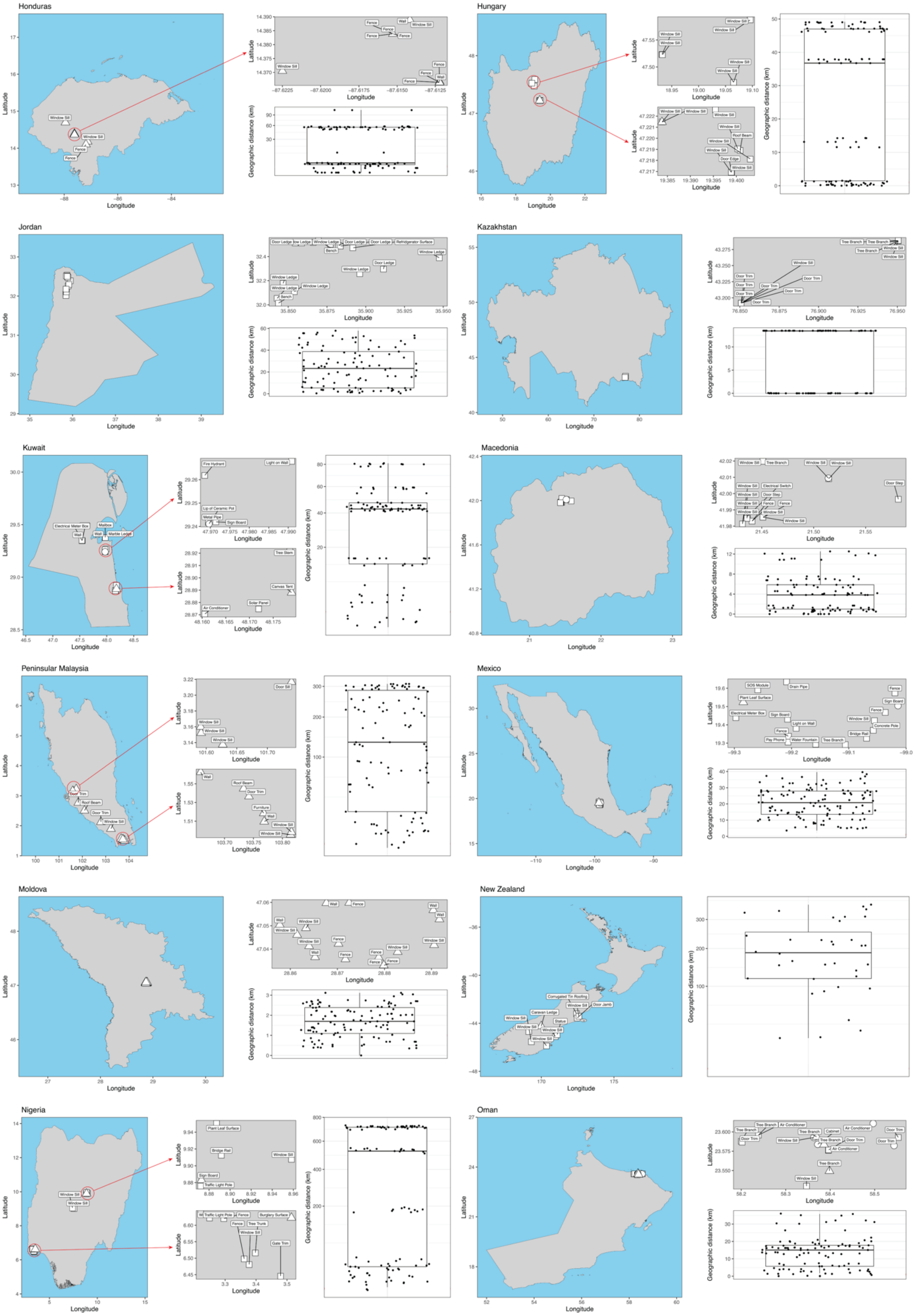

Honduras
Hungary
Jordan
Kazakhstan
Kuwait
Macedonia
Peninsular Malaysia
Mexico
Moldova
New Zealand
Nigeria
Oman
Latitude
Longitude
Geographic distance (km)

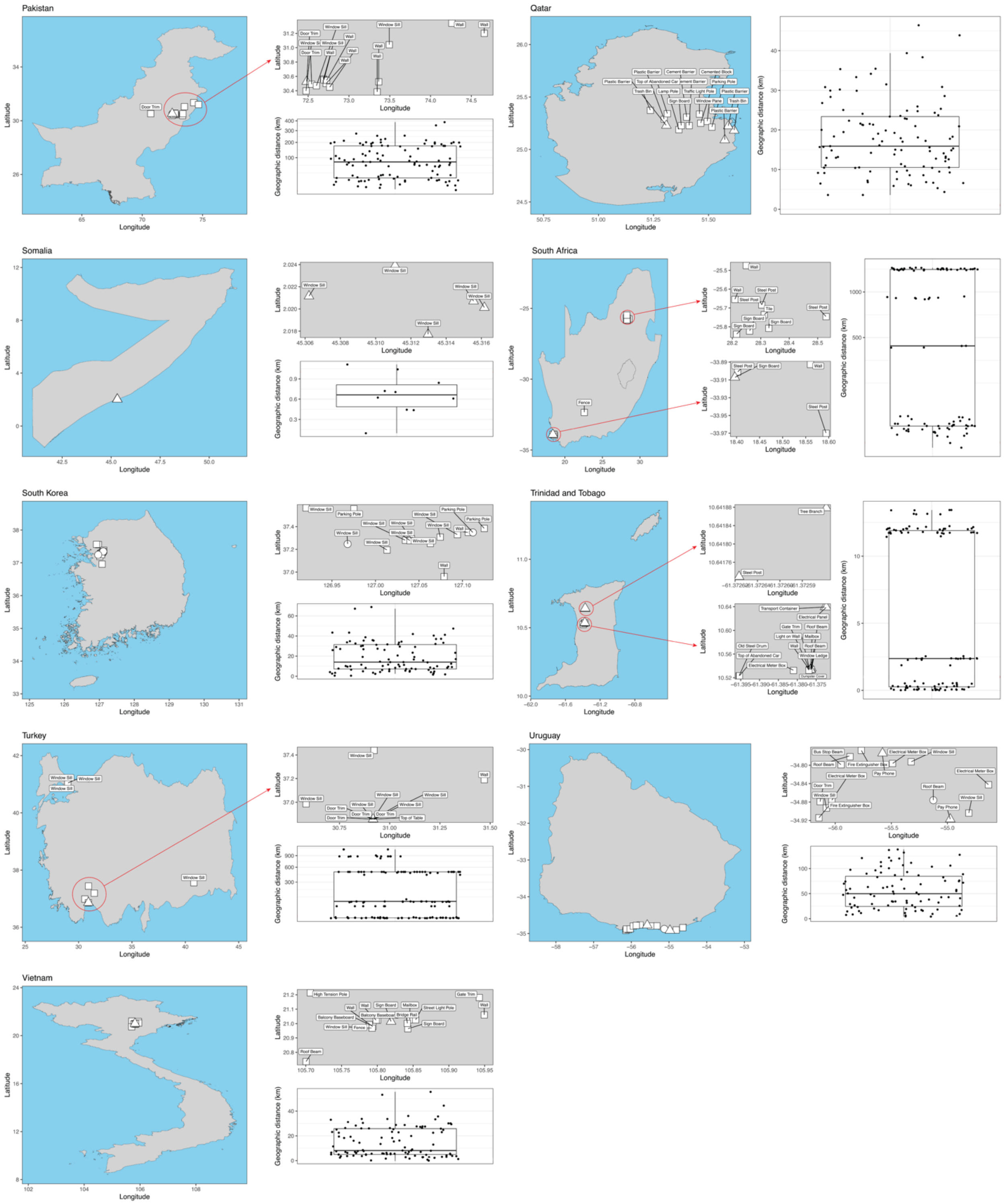

Figure S1. Geographic distribution of dust samples within each country. A total of 467 dust samples were collected from 33 countries. Most of the dust samples are collected from window sills (150 samples), door trims (46 samples), walls (35 samples), and fences (30 samples). Square symbols denote samples having both bacterial and fungal data, circle symbols denote samples having only bacterial data, and triangle

symbols denote samples having only fungal data. A zoomed view of the geographic distribution of dust samples is shown if it is unclear in the country map. Boxplots show the pairwise geographic distances between dust samples, data points are jittered to enhance clarity.

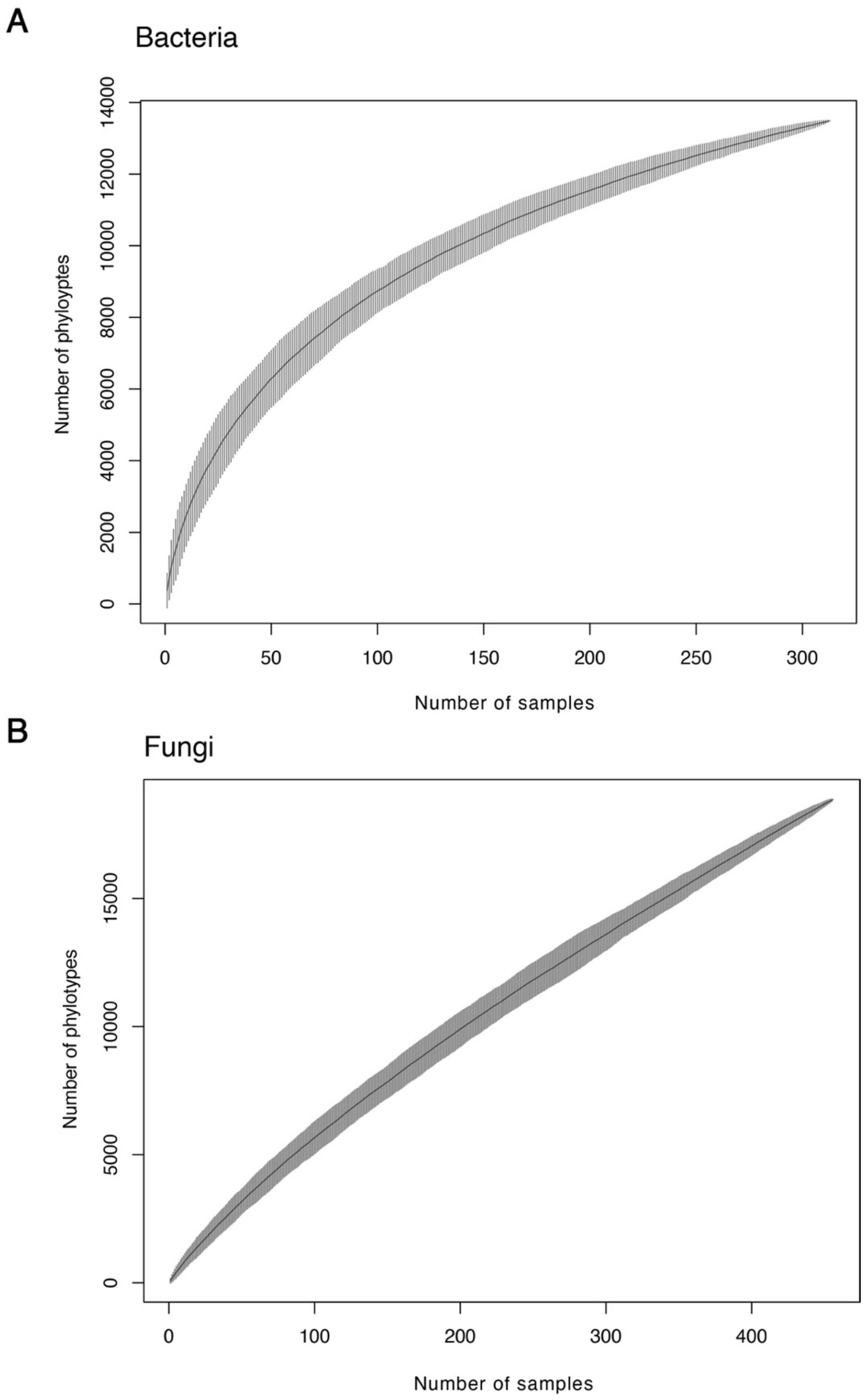

Figure S2. Phylotype accumulation curves.

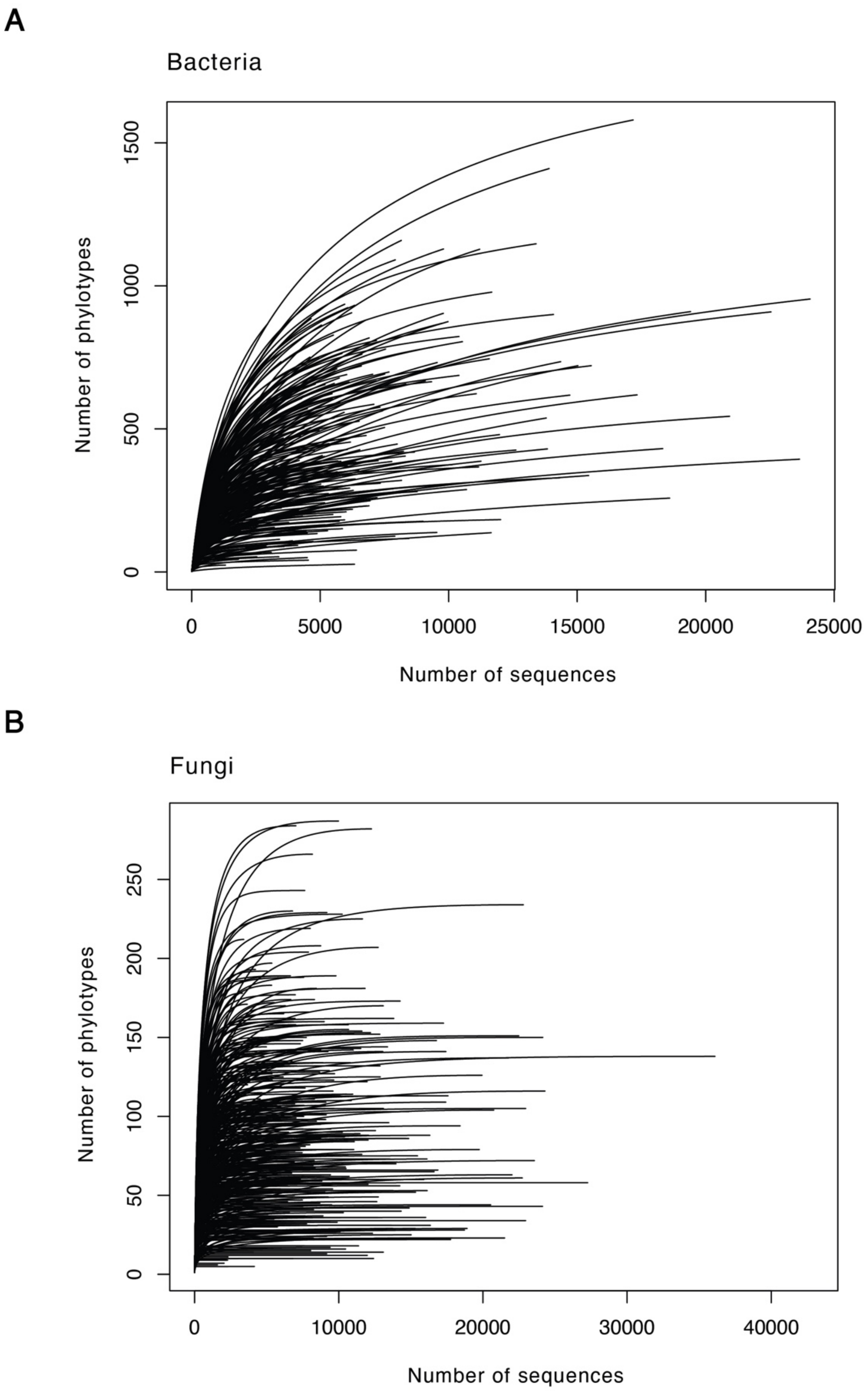

Figure S3. Rarefaction curves.

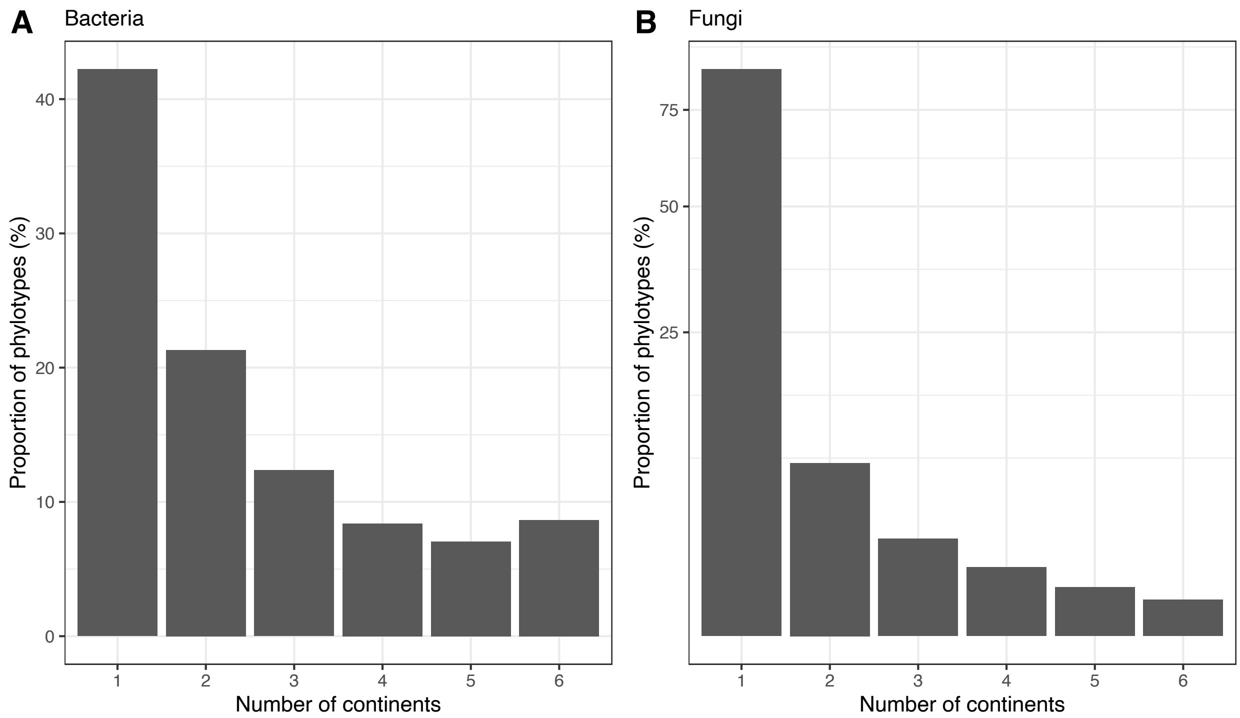

Figure S4. Continental occupancy pattern of phylotypes. The proportions of phylotypes detected in one, two, three, four, five, and six continents are shown.

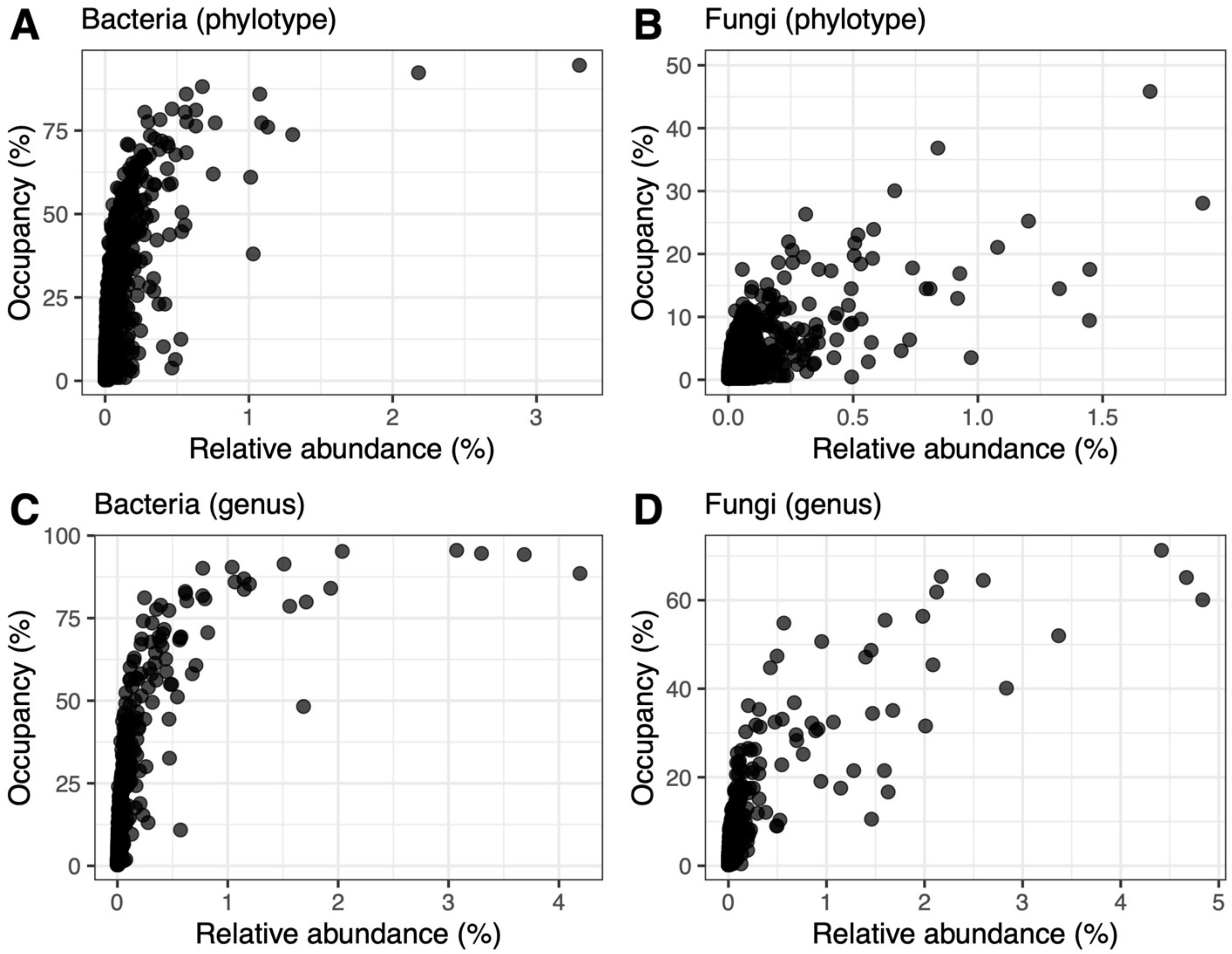

Figure S5. Occupancy-abundance relationship. The relationship between occupancy (proportion of samples) and relative abundance at the phylotype (A and B) and genus levels (C and D).

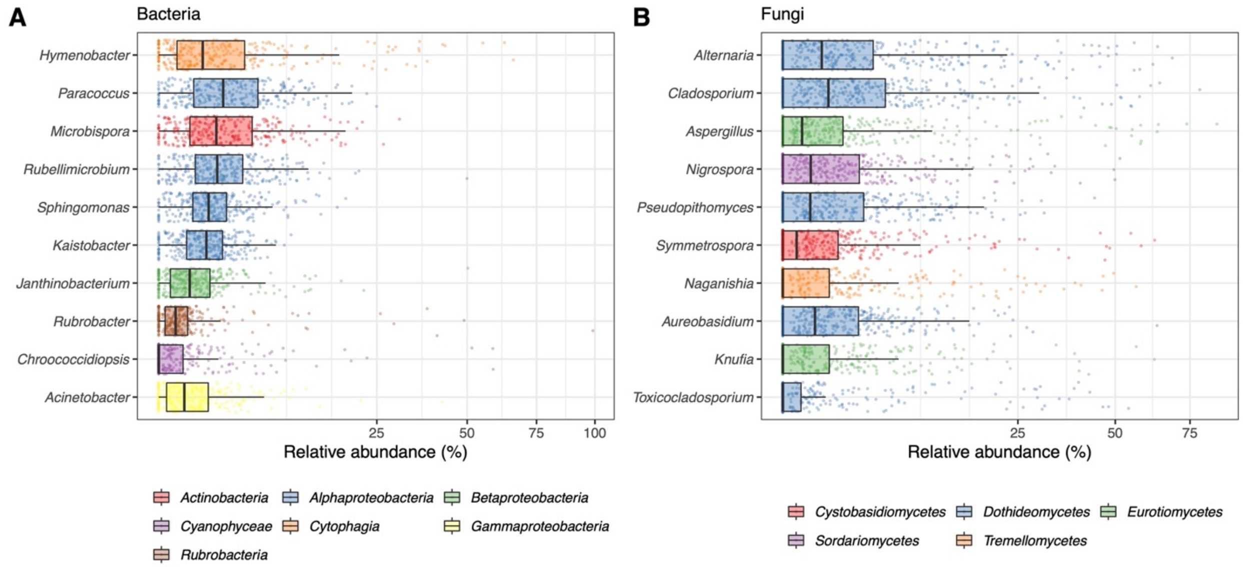

Figure S6. Dominant microbial lineages at the genus level. The top 10 most dominant bacterial genera (A) and fungal genera (B) are shown.

Table S1 Results of variation partitioning

	Bacteria	Fungi
Unique effect of environmental factors	9.90%	10.94%
Unique effect of geographic distance	1.34%	2.92%
Joint effect of environmental factors and geographic distance	2.97%	2.21%

Table S2 Climatic and soil variables in this study.

Category	Variable	Database
Climate	**Mean annual temperature**	WorldClim
	Mean diurnal range	
	Isothermality	
	Temperature seasonality	
	Max temperature of the warmest month	
	Min temperature of the coldest month	
	Temperature annual range	
	**Mean temperature of the wettest quarter**	
	Mean temperature of the driest quarter	
	Mean temperature of the warmest quarter	
	Mean temperature of the coldest quarter	
	**Temperature of the sampling month**	
	**Mean annual precipitation**	
	Precipitation of the wettest month	
	**Precipitation of the driest month**	
	Precipitation seasonality	
	Precipitation of the wettest quarter	
	Precipitation of the driest quarter	
	Precipitation of the warmest quarter	
	**Precipitation of the coldest quarter**	
	**Precipitation of the sampling month**	
Soil	**Bulk density**	Harmonized World Soil Database
	Base saturation	
	**Organic carbon**	
	**pH**	

Variables in bold were retained after multicollinearity examination (variance inflation factor < 5).
